# Predicting Lyme Disease: A One Health Approach

**DOI:** 10.3390/pathogens15040393

**Published:** 2026-04-07

**Authors:** Mollie McDermott, Shamim Sarkar, Janice O’Brien, Karen Gruszynski, Barbara Shock, Vina Faulkner, Lauren Wisnieski

**Affiliations:** 1 Richard A. Gillespie College of Veterinary Medicine, Lincoln Memorial University, Harrogate, TN 37752, USA; mollie.mcdermott@lmunet.edu (M.M.); shamim.sarkar@lmunet.edu (S.S.); kgrusz@midwestern.edu (K.G.); vina.faulkner@lmunet.edu (V.F.); 2 Population Health Sciences Department, Virginia-Maryland College of Veterinary Medicine, Virginia Tech, 205 Duck Pond Drive, Blacksburg, VA 24061, USA; janiceobrien@vt.edu; 3 College of Graduate Studies, Midwestern University, 555 31st Street, Downers Grove, IL 60515, USA; 4 College of Mathematics, Sciences, and Health Professions, Lincoln Memorial University, Harrogate, TN 37752, USA; barbara.shock@lmunet.edu

**Keywords:** Lyme disease, one health, Google Trends, environment, canine, predictive modeling, tick-borne diseases

## Abstract

Lyme disease is the most common vector-borne disease in North America. Predicting Lyme disease incidence is a key component of public health preparedness. Previously, we demonstrated that the volume of data searches on Google Trends for terms related to Lyme disease, such as “Lyme” and “tick bite”, can be used as a tool to predict monthly human Lyme disease incidence at the state level. The objective of this project was to build upon our previous work by adding environmental and canine data to our predictive models for the prediction of state-level human and canine Lyme disease incidence. Human data were acquired from state health departments. Canine data were acquired from IDEXX Laboratories. We hypothesized that incorporating a One Health approach with human, animal, and environmental data would improve the predictive ability of the models. The One Health model performed significantly better (Mean Absolute Error [MAE] = 12.1) in predicting human disease incidence in 6 out of 16 states compared to the environmental data model (MAE = 16.5), human search terms model (MAE = 21.4), canine data (search terms + case count) model (MAE = 31.1), and the canine case data model (MAE = 32.0). For canine Lyme disease incidence, the One Health model performed worse (MAE = 330.5) compared to the canine search data model (MAE = 282.3), the human data (search terms + cases) model (MAE = 248.4), and the environmental data (MAE = 221.5) model. However, even the best-performing models had large prediction errors, which limit practical utility. Future studies should incorporate alternative data streams, such as electronic health records and insurance claims, to test predictive ability.

## 1. Introduction

Lyme disease is caused by infection with spirochete bacteria within the *Borrelia burgdorferi* sensu lato (*B. burgdorferi* s.l.) complex, where *Borrelia burgdorferi* sensu stricto is the primary pathogen in human and animal populations in North America. In the *B. burgdorferi* s.l. complex, *B. mayonii* was identified as a novel human pathogen in 2016 when discovered in Minnesota and Wisconsin residents [1]. It is likely that additional *Borrelia* spp., including those in the Bbsl complex, will be discovered with increased surveillance and advances in next-generation screening of humans, domestic animals and wildlife [2].

Lyme disease is an increasingly significant public health issue across the eastern United States (U.S.). In 2023, over 89,000 Lyme disease cases were reported through routine national surveillance, but alternative estimates suggest that as many as 476,000 people are treated for Lyme disease annually in the U.S. [3]. Once largely confined to the Northeast, Lyme disease has expanded northward into Canada, westward into Ohio, Iowa, and Illinois, and has become a growing concern in Virginia and the American Southeast associated with the range expansion of the eastern blacklegged tick, *Ixodes scapularis* [4]. The western blacklegged tick, *Ixodes pacificus*, is the vector primarily responsible for spreading Lyme disease throughout the western portion of the U.S.

The continued increase in Lyme disease incidence has been attributed to climate change, increased humidity, and human sprawl, which have pushed wildlife into increasingly anthropogenic landscapes [5]. While white-tailed deer (*Odocoileus virginianus*) are important reproductive hosts for adult ticks, several small mammal reservoir species play a critical role in maintaining and transmitting *B. burgdorferi* s.l. In the northeast, the white-footed mouse (*Peromyscus leucopus*) is a key reservoir host, whereas in western U.S., species such as the western gray squirrel (*Sciurus griseus*) and dusky-footed woodrat (*Neotoma fuscipes*) contribute to pathogen maintenance in tick populations [6,7]. These species readily occupy peridomestic and human-altered environments, increasing opportunities for infected nymphal ticks to encounter humans and domestic animals.

Climate change has further contributed to tick range expansion by altering the distribution and seasonal activity of *Ixodes* species. Notably, behavioral differences between northern and southern blacklegged tick populations may influence host-seeking patterns and, ultimately, disease transmission dynamics [8]. Key environmental factors, such as mild winters, humid summers, reduced burning regimes, and dense vegetation, play critical roles in preventing tick desiccation and facilitating the persistence of *B. burgdorferi* s.1 [9]. Together, these environmental changes, shifting land use patterns, and expanding vector and reservoir host communities have transformed Lyme disease from a historically regional issue into a pressing One Health challenge [5,10].

The *Borrelia burgdorferi* sensu lato (s.l.) complex comprises at least 28 genospecies, including seven “Candidatus” taxa, recently proposed to be split into two genera: *Borreliella* and *Borrelia* [11]. Of the 18 genospecies for which pathogenic potential has been evaluated, 11 are found only in Eurasia, with *B. afzelii* and *B. garinii* identified as human pathogens in addition to *B. burgdorferi* s.s. [12]. In North America, *B. burgdorferi* s.s. and, more rarely, *B. mayonii* are human pathogens, and the roles of other genospecies as human or canine pathogens remain poorly characterized. Climate change has altered the phenology of *Ixodes* spp., which transmit Lyme disease throughout Eurasia and follow a life cycle similar to that of *Ixodes* spp. in North America [13]. In the U.S., diagnostic tests for Lyme disease predominantly use the *B. burgdorferi* B31 strain, whereas multiple Bbsl strains contribute to Lyme disease surveillance in Eurasia. Our study focuses exclusively on North American diagnostic data, so these differences do not affect model development or evaluation.

The volume of search trends in the Google Trends data report by geographic area (metropolitan area and state) has been used for prediction of various infectious diseases, including Lyme disease [14], influenza [15], and Zika [16]. Building on our prior work [17], which demonstrated the potential utility of Google Trends data for predicting monthly Lyme disease incidence at the state level, the present study extends this approach by integrating environmental variables and canine case data to improve prediction accuracy and explore a One Health perspective. Canine populations serve as valuable sentinels for human Lyme disease risk [18,19]. Rising seroprevalence in dogs has been correlated with increased human cases [20]. This study further advances the methodology by evaluating model performance using bootstrapped Mean Absolute Error (MAE) and Mean Absolute Percentage Error (MAPE) with uncertainty quantification, and by comparing predictive patterns across both human and canine populations, which were not assessed in our previous work. We hypothesized that incorporating environmental and canine data to address the One Health triad would improve predictive accuracy for state-level Lyme disease incidence. In the U.S., Lyme disease surveillance and reporting are conducted primarily at the state level by health departments and the CDC, making this a relevant scale for public health decision-making. Ultimately, findings from this work are intended to inform more timely and geographically responsive Lyme disease surveillance and prevention strategies by supporting early risk detection, guiding public health messaging, and helping veterinary and medical communities anticipate periods of elevated exposure risk.

## 2. Materials and Methods

This ecological study was approved by the Lincoln Memorial University Institutional Review Board (IRB #2025/3/1).

### 2.1. Data Sources

#### 2.1.1. Lyme Disease Data

Monthly, state-level human Lyme disease case counts were collected through publicly available online repositories and direct requests to state health departments [21,22,23,24]. Specific data notes from health departments, where applicable, are in Supplementary File. States reporting fewer than 10 cases annually were excluded to reduce instability associated with very small case counts and protect the privacy and identity of individual cases, as small cell sizes can make individuals potentially identifiable while also producing unreliable rate estimates and disproportionate influence of random variation on model performance. Final inclusion was determined based on data completeness and availability. The states included for analysis were California, Connecticut, Indiana, Kansas, Maine, Michigan, North Dakota, New Hampshire, Oregon, Rhode Island, South Carolina, Texas, Virginia, Vermont, Washington, and West Virginia. Canine Lyme disease test results (SNAP™ 4Dx™ Test or SNAP™ 4Dx™ Plus Test) from 2010 to 2019 were obtained from IDEXX Laboratories, Inc. (Westbrook, ME, USA).

#### 2.1.2. Google Trends Data

The search volume data from Google Trends were retrieved using the gtrendsR package in R version 4.0.2 [25]. Google Trends reports relative interest scores from 0 to 100, with 100 representing peak popularity during the queried time frame and region [26]. Term selection was informed by prior research, a literature review, and input from subject matter experts. Based on results from our previous study [17], we chose the two top-performing search terms for predicting human Lyme disease cases: “Lyme disease” and “Lymes” for inclusion in the predictive models presented in this study.

To capture search interest in canine Lyme disease, we included search terms related to dog-specific symptoms, tick-borne diseases, and canine health that would be applicable to the general public. To account for variation in phrasing, we included both direct and reversed word orders (e.g., “dog limping” and “limping dog”) when there was reportable search volume for those terms. The final search terms included:

• Lyme disease and vector-related terms: “dog Lyme”, “Lyme dog”, “tick dog”.
• Clinical signs and symptoms: “dog limping”, “limping dog”, “dog lethargic”, “lethargic dog”, “not eating dog”.
• Physical abnormalities: “bump dog”, “lump dog”, “dog lump”, “dog swelling”, “swelling dog”.
• Chronic and infectious diseases: “dog arthritis”, “arthritis dog”, “kennel cough”, “distemper”, “dog cancer”, “cancer dog”.

Each term was independently evaluated for its predictive performance against state-level human canine disease case counts using MAE. The two terms with the lowest error, indicating the strongest model fit, were retained for inclusion in the final predictive models used in the analysis.

#### 2.1.3. Environmental Data

Environmental data were aggregated at the monthly state-level to align with outcome measures. Environmental data, including the monthly maximum and minimum temperatures, total precipitation (inches), and average humidity (%), were obtained from the NOAA National Centers for Environmental Information [27].

### 2.2. Statistical Analysis

Descriptive statistics (mean, standard deviation, minimum, median, and maximum) for monthly state-level canine Lyme disease cases were summarized by month and year. Descriptive statistics for monthly state-level human Lyme disease cases were presented in a study by Wisnieski et al., 2023 [17].

To forecast monthly, state-level human and canine Lyme disease case counts, the data were structured as state-month observations (“xtset” state time). Twelve-month expanding window negative binomial regression models were estimated using the “rolling” command to generate state-level forecasts while accounting for both temporal trends and differences across states in Stata version 19.0 [28]. For each month, the model was trained on the preceding 12 months of data, including lagged incidence, Google search term volumes, and climate variables, and then used to predict the subsequent month’s cases. Coefficients were updated recursively as new data became available, allowing the model to generate out-of-sample forecasts while using only information that would have been available at the time of prediction. This approach ensures that predictions reflect a realistic forecasting scenario rather than retrospective fitting. A negative binomial framework was selected after identifying over-dispersion in the outcome data (i.e., human and canine Lyme disease case counts).

We aimed to compare the predictive performance of models incorporating data from components of the One Health Triad: human health, animal health, and environmental factors. By evaluating models that included data from each domain individually, as well as in combination, we were able to assess the relative and combined contributions of these data sources to predict human Lyme disease cases. Each variable in the models included a 12-month lag to account for seasonal differences, allowing us to more accurately capture temporal patterns in disease dynamics. We used the following configurations for models predicting monthly state-level human Lyme disease case counts:

1. Human search terms (search volume for “Lyme disease” and “Lymes”);
2. Canine data (search volume for “tick dog” and “Lyme dog” + case counts);
3. Canine case counts *;
4. Environmental data (maximum and minimum temperatures, precipitation, and average humidity);
5. One Health (volume of human search terms for “Lyme disease” and “Lymes”] + canine case counts + environmental data [maximum and minimum temperatures, precipitation, and average humidity]).

* Note that for the human configurations, one model included only canine case data (without canine search data), because canine search term data were missing for certain low-volume states (Kansas, North Dakota, and Vermont) due to data suppression in Google Trends for low search frequency and the inclusion of search terms in the model had a low impact on the results. The One Health model excluded canine search data in order to maximize available sample size and generalizability to low search volume states.

For the monthly state-level canine Lyme disease models, we used the following configurations:

1. Human data (search term volume for “Lyme disease” and “Lymes” + case counts);
2. Canine search terms (search volume for “tick dog” and “Lyme dog”);
3. Environmental data (maximum and minimum temperatures, precipitation, and average humidity);
4. One Health data: (volume of human search terms volume for “Lyme disease” and “Lymes” + human case counts + canine search term volume for “tick dog” and “Lyme dog” + environmental data [maximum and minimum temperatures, precipitation, and average humidity])

Model performance was assessed by calculating the MAE and the Mean Absolute Percentage Error (MAPE) for each model configuration. MAE reflects the average deviation between predicted and observed Lyme disease case counts. MAPE reflects the mean of the absolute differences between predicted and observed incidence, divided by the observed incidence, expressed as a percentage. To quantify uncertainty, we generated bootstrapped estimates of MAE and corresponding 95% confidence intervals (CIs) using 1000 resamples. Comparisons between models were made by examining the degree of overlap in the CIs for MAEs: non-overlapping CIs were interpreted as indicating a statistically significant difference in predictive performance, while overlapping CIs suggested no significant difference between models. The canine and One Health models for the three states are not included due to missing canine search term data, as described above.

## 3. Results

Data were obtained from 16 U.S. states. A map of the states with high incidence state designations and descriptive statistics for human Lyme disease cases is presented in the study by Wisnieski et al. 2023 [17].

### 3.1. Descriptive Statistics

Missing data for the volume of monthly search terms assessed for overall completeness across 16 states and 10 years. “Tick dog” was noted to be the most complete search term with 0 missing values. The least complete terms were “dog cancer” and “cancer dog”, which had 1800 missing values (93.8% missing). There were moderate levels of completion with other terms of interest, such as “dog Lyme” and “dog swelling”, that were missing approximately 25% and 50% of values, respectively.

Descriptive statistics for canine Lyme disease cases are presented in Table 1 and Table 2. Canine cases peaked during the months of March to June, evidenced by means > 400 for canine cases per month among the 16 states.

### 3.2. Human Lyme Disease Models

**Figure 1:**
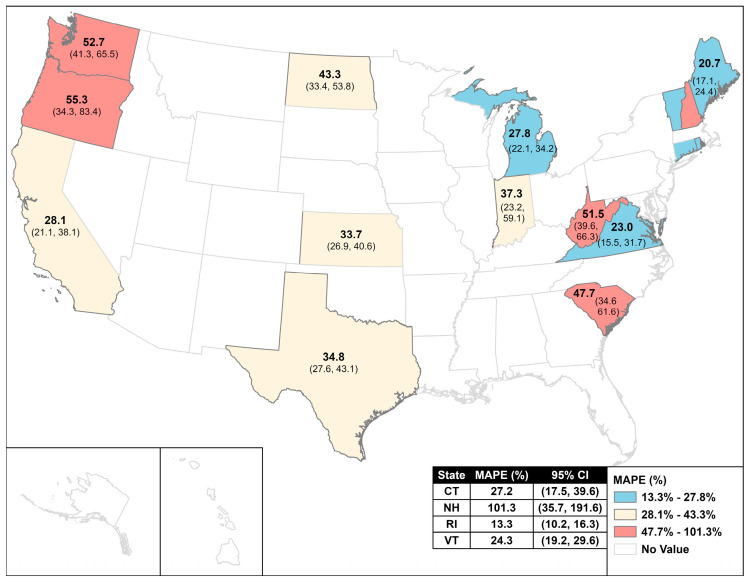
Mean Absolute Percentage Error (MAPE) for a predictive model of human Lyme disease case counts using a One Health data stream for 16 states in the United States. The One Health data stream incorporates human Lyme Google search terms, canine Lyme case counts, and environmental data.

The results of the predictive models for human Lyme disease are presented in Table 3 and Table 4. Overall, the One Health model performed better than models with only one component of the One Health triad (Table 3). However, prediction errors were high. The average MAPE for the One Health model was 39.7% (95%: 33.8, 47.7) (Table 4). When stratified by state, the One Health model was significantly better than all other models in 6 out of 16 states. To illustrate variation across the U.S., MAPEs across the U.S. are displayed in Figure 1. In seven states, the One Health model performed equally as well as the environmental model, but better than the human search term model and the canine data models (Table 3). In two states (Oregon and Washington), there were no differences between any of the models.

### 3.3. Canine Lyme Disease Models

The results of the predictive models for canine Lyme disease case counts are presented in Table 5 and Table 6. Due to the low canine search term volume, only 13 out of 16 states were included in the canine search term and One Health models. Overall, the models for canine Lyme disease performed extremely poorly, with large MAPEs (Table 6). The One Health model performed worse than the models with human data, canine search terms, and environmental data. When stratified by state, the One Health model performed worse in 8 out of 13 states. There were no significant differences in model performance between the five states (Connecticut, Kansas, Oregon, Vermont, and Washington). The environmental data model performed best in California and North Dakota.

## 4. Discussion

In this study, predictive models for human and canine Lyme disease incidence were built using One Health (human, canine, and environmental) data. We hypothesized that including One Health data would improve model predictions based on prior research findings that emphasized the need for incorporation of One Health surveillance systems for zoonotic diseases [29,30]. However, inclusion of One Health data only improved human Lyme disease predictions in 6 out of 16 states and did not improve canine Lyme disease predictions. Overall, the models had large prediction errors and alternative prediction models are needed to accurately forecast Lyme disease incidence.

Similar studies had varying results. In the study by O’Brien et al. (2025), including canine Lyme disease insurance claims in predictive models did not improve prediction of human Lyme incidence [31]. By contrast, Bouchard et al. (2023) found an association between human Lyme disease cases and risk maps based on Lyme disease knowledge and behavior and ecological components of Lyme disease [32]. The predictive ability of a One Health model depends on the type, quality, and operability of the data with other data sources [29]. In our study, we utilized Lyme disease case information from state health departments, which is a passive surveillance system that underreports the true number of cases [33,34]. In addition, surveillance systems vary by state. To ease the burden of case reporting, case definitions do not require clinical information in high-incidence states, but this information is required in low-incidence states [34]. Our models could potentially be strengthened using other metrics for human Lyme disease incidence in addition to state health department data, such as insurance claims or electronic health records [35,36]. In addition, the use of Google Trends data for some search terms was limited in some states, leading to the inability to produce One Health models for canine Lyme disease incidence in three states. Careful consideration of applicable search terms that have an adequate search volume is imperative for future research [17]. Lastly, human Lyme disease case data and canine IDEXX results represent distinct surveillance sources with different reporting mechanisms and potential biases. Human cases are derived from passive public health surveillance, whereas canine test data reflect veterinary diagnostic testing patterns. As such, these datasets should be interpreted as complementary indicators of Lyme disease risk rather than directly equivalent measures of incidence. These differences in data generation and reporting may also contribute to heterogeneity in model predictive performance between the human and canine outcomes.

### 4.1. Limitations

There were several limitations that affected this study. Our analysis was conducted at the state scale to align with the spatial resolution of certain predictors, including Google Trends data, which are reported only at state or metropolitan levels. The use of state-level disease incidence and climate variables, aggregated at a monthly resolution, may have influenced model performance and obscured within-state heterogeneity in environmental conditions, tick ecology, and Lyme disease risk. A more refined analysis using county-level incidence and alternative data sources could provide more precise, spatially relevant estimates. In addition, we included data from only 16 states, which limits the generalizability of the model to other U.S. regions and Canadian provinces.

Because of the limited geographic scope, we were also unable to stratify analyses by major ecological regions (e.g., eastern, Midwestern, and western coastal U.S.) that differ in tick vector species, particularly *Ixodes scapularis* and *Ixodes pacificus*. These species exhibit important ecological and behavioral differences that may influence transmission dynamics and model performance under varying environmental conditions. Future studies incorporating broader geographic coverage could evaluate whether predictive relationships differ across vector regions and how shifting tick distributions associated with climate change may alter these dynamics [4,7,9].

Expanding future models to include additional states and Canadian provinces, along with more spatially resolved disease surveillance data, could strengthen generalizability and improve our understanding of regional variation in Lyme disease risk.

### 4.2. Conclusions

Overall, the inclusion of One Health data improved prediction of human Lyme disease incidence in some states but did not improve prediction of canine Lyme disease incidence. However, even the best-performing models exhibited substantial prediction errors, limiting their practical utility. We recommend testing other data streams to produce higher-performing predictive models, such as electronic health record data and other environmental data. Future studies can also consider more refined analyses by predicting county-level disease incidence.

## Figures and Tables

**Table 1:** Descriptive statistics for canine Lyme disease cases by month for 16 U.S. states over 10 years (2010–2019).

Month	Mean	SD	Minimum	Median	Maximum
January	300.51	403.9	0	111.0	1708
February	334.1	464.0	0	109.0	2158
March	439.3	595.0	0	157.0	2306
April	486.6	620.2	0	153.0	2597
May	488.3	630.0	0	188.0	2717
June	457.9	606.1	0	159.0	2755
July	393.6	504.7	0	148.0	2108
August	297.4	422.1	0	111.0	1888
September	246.2	325.8	0	112.0	1448
October	273.0	357.6	0	123.0	1661
November	258.3	339.4	0	98.0	1600
December	235.7	299.2	0	112.0	1413

**Table 2:** Descriptive statistics for canine Lyme disease cases by year (2010–2019) for 16 U.S. states over 10 years.

Year	Mean	SD	Minimum	Median	Maximum
2010	134.7	189.7	0	29.5	806
2011	182.9	254.9	0	59.5	991
2012	238.2	331.5	0	80.5	1249
2013	264.2	362.1	0	91.5	1426
2014	324.9	445.0	0	118.5	1867
2015	367.5	501.1	0	142.5	1872
2016	434.1	597.6	0	178.5	2491
2017	488.7	657.3	1	218.5	2755
2018	505.6	651.2	1	262.0	2732
2019	566.31	681.9	2	317.5	2670

**Table 3:** Mean Absolute Error (MAE) and 95% bootstrapped CI of predictive models for monthly state-level human Lyme disease case count (N = 1920).

State	Human Search Data	Canine Data (Search Terms + Case Counts)	Canine Case Data	Environmental Data	One Health Data ^1^
CA	4.5 (3.7, 5.4) ^a^	4.5 (3.7, 5.3) ^b^	4.9 (4.1, 5.8) ^c^	2.8 (2.2, 3.5) ^a^	2.4 (1.9, 3.0) ^d^
CT	56.0 (41.6, 71.6) ^a^	109.6 (93.1, 126.8) ^b^	134.2 (115.6, 153.8) ^c^	61.7 (48.4, 76.2) ^a^	34.7 (25.1, 46.4) ^d^
IN	4.4 (3.2, 5.7) ^a^	6.7 (4.3, 9.6) ^ab^	10.3 (8.1, 12.9) ^b^	4.4 (3.2, 5.7) ^a^	2.6 (1.9, 3.5) ^c^
KS	1.5 (1.2, 1.8) ^a^	-	1.8 (1.5, 2.0) ^a^	1.1 (0.9, 1.4) ^b^	0.9 (0.7, 1.1) ^b^
ME	70.5 (55.1, 88.4) ^a^	69.1 (53.7, 85.6) ^a^	78.0 (63.2, 94.9) ^a^	36.4 (27.1, 47.7) ^b^	28.9 (20.5, 38.2) ^b^
MI	10.9 (7.7, 14.8) ^a^	13.2 (8.6, 18.9) ^a^	13.9 (11.5, 16.7) ^a^	7.3 (4.9, 10.1) ^b^	3.6 (2.6, 4.8) ^b^
ND	2.2 (1.5, 3.1) ^a^	-	2.6 (2.1, 3.1) ^a^	1.2 (0.9, 1.6) ^b^	1.0 (0.7, 1.3) ^b^
NH	45.7 (35.1, 57.2) ^a^	64.1 (54.0, 76.4) ^b^	72.0 (59.7, 85.0) ^b^	38.3 (29.4, 47.8) ^a^	29.2 (21.4, 37.2) ^c^
OR	17.2 (13.5, 21.0) ^a^	15.5 (12.4, 18.6) ^a^	17.2 (14.4, 19.9) ^a^	15.1 (11.8, 18.9) ^a^	13.1 (9.7, 16.4) ^a^
RI	21.3 (15.1, 28.2) ^a^	37.6 (30.6, 45.1) ^b^	45.5 (38.0, 53.6) ^c^	17.9 (13.1, 23.3) ^a^	9.9 (7.2, 13.2) ^d^
SC	1.8 (1.5, 2.1) ^a^	1.9 (1.6, 2.2) ^a^	1.9 (1.6, 2.3) ^a^	1.6 (1.3, 1.8) ^b^	1.5 (1.2, 1.8) ^b^
TX	2.6 (1.7, 2.8) ^a^	2.0 (1.6, 2.5) ^a^	2.8 (2.2, 3.3) ^a^	1.7 (1.3, 2.2) ^b^	1.5 (1.1, 1.8) ^b^
VA	44.2 (35.3, 54.8) ^a^	38.7 (31.5, 46.6) ^a^	44.8 (37.7, 53.1) ^a^	27.5 (21.0, 34.5) ^b^	22.4 (14.8, 31.3) ^b^
VT	31.8 (23.9, 41.2) ^a^	-	43.0 (34.6, 52.5) ^b^	21.6 (15.9, 28.3) ^c^	14.1 (10.1, 18.5) ^d^
WA	1.7 (1.3, 2.0) ^a^	2.1 (1.4, 3.2) ^a^	2.1 (1.7, 2.6) ^a^	1.3 (1.0, 1.6) ^a^	1.2 (1.0, 1.5) ^a^
WV	19.6 (14.3, 25.6) ^ab^	26.4 (19.2, 35.6) ^b^	30.8 (22.9, 40.1) ^b^	19.1 (12.3, 26.9) ^ab^	16.2 (10.8, 22.5) ^a^
Overall	21.4 (19.2, 24.0) ^a^	31.1 (28.0, 34.2) ^b^	32.0 (29.3, 35.1) ^b^	16.5 (14.7, 18.4) ^c^	12.1 (10.5, 13.5) ^d^

Within a row, values sharing the same superscript letter are not significantly different (*p* > 0.05). ^1^ This model included human search terms + canine case counts + environmental data.

**Table 4:** Mean Absolute Percentage Error (MAPE) and 95% bootstrapped CI of predictive models for monthly state-level human Lyme disease case count (N = 1920).

	MAPE (%) (95% CI)				
State	Human Search Data	Canine Data (Search Terms + Case Counts)	Canine Case Data	Environmental Data	One Health Data
CA	54.1 (42.0, 70.7)	57.8 (44.6, 72.5)	63.4 (48.8, 81.3)	32.0 (24.0, 41.0)	28.1 (21.1, 38.1)
CT	45.0 (29.3, 65.9)	112.7 (78.7, 159.1)	147.8 (104.7, 200.8)	51.2 (35.0, 71.3)	27.2 (17.5, 39.6)
IN	51.9 (33.0, 80.0)	174.0 (55.3, 397.7)	263.8 (157.4, 404.4)	49.5 (36.7, 68.5)	37.3 (23.2, 59.1)
KS	46.6 (40.6, 53.3)	-	57.5 (43.6, 67.6)	39.1 (33.5, 45.6)	33.7 (26.9, 40.6)
ME	68.9 (54.3, 85.8)	67.5 (54.5, 82.1)	107.6 (83.8, 132.8)	25.5 (22.2, 29.1)	20.7 (17.1, 24.4)
MI	75.8 (57.7, 98.7)	92.4 (68.4, 121.3)	238.2 (173.7, 310.2)	43.6 (37.8, 50.2)	27.8 (22.1, 34.2)
ND	65.3 (48.8, 86.8)	-	79.9 (63.6, 97.9)	48.4 (38.0, 60.7)	43.3 (33.4, 53.8)
NH	138.5 (56.7, 2.52)	225.3 (108.4, 375.2)	247.1, 126.8, 402.0)	141.3 (54.5, 250.0)	101.3 (35.7, 191.6)
OR	220.9 (69.2, 461.1)	130.7 (71.7, 207.7)	180.3 (95.6, 284.2)	79.7 (48.5, 116.0)	55.3 (34.3, 83.4)
RI	23.0 (18.9, 26.7)	60.4 (48.3, 71.9)	79.4 (62.3, 99.6)	20.7 (17.0, 24.2)	13.3 (10.2, 16.3)
SC	55.5 (46.0, 66.7)	70.8 (53.4, 89.0)	68.5 (52.4, 86.2)	45.4 (35.6, 57.5)	47.7 (34.6, 61.6)
TX	63.6 (45.7, 84.5)	57.3 (43.3, 73.1)	86.3 (59.4, 114.6)	43.7 (33.8, 55.9)	34.8 (27.6, 43.1)
VA	74.2 (48.3, 107.6)	100.3 (56.6, 154.7)	131.3 (69.8, 219.8)	36.1 (27.7, 45.1)	23.0 (15.5, 31.7)
VT	57.6 (44.9, 73.3)	-	125.2 (97.6, 155.9)	30.9 (26.5, 35.7)	24.3 (19.2, 29.6)
WA	69.9 (50.7, 94.3)	130.9 (61.2, 258.3)	90.0 (68.5, 114.4)	53.7 (43.2, 66.3)	52.7 (41.3, 65.5)
WV	82.6 (60.0, 109.5)	108.1 (78.9, 142.7)	144.5 (104.3, 190.8)	45.0 (37.7, 56.5)	51.5 (39.6, 66.3)
Overall	74.7 (57.3, 98.3)	107.7 (90.4, 130.2)	136.6 (112.8, 163.3)	49.9 (4.1, 39.0)	39.7 (33.8, 47.7)

**Table 5:** Mean Absolute Error (MAE) and 95% bootstrapped CI of predictive models for monthly state-level canine Lyme disease case count (N = 1920).

State	Human Data (Search Terms + Cases)	Canine Search Terms	Environmental Data	One Health Data ^1^
CA	94.9 (92.0, 98.0) ^a^	94.8 (91.4, 98.3) ^a^	91.6 (89.1, 94.0) ^b^	97.8 (94.1, 101.7) ^a^
CT	831.9 (754.5, 908.2) ^a^	852.1 (765.2, 939.1) ^a^	784.7 (728.7, 845.2) ^a^	876.7 (792.6, 977.6) ^a^
IN	178.1 (163.7, 193.4) ^a^	130.4 (116.2, 145.6) ^b^	116.3 (105.7, 128.4) ^b^	207.3 (188.7, 228.0) ^c^
KS	3.1 (2.8, 3.4) ^a^	-	3.1 (2.8, 3.5) ^a^	-
ME	643.2 (595.3, 692.1) ^a^	700.5 (628.2, 776.1) ^a^	642.8 (594.0, 692.2) ^a^	756.6 (682.7, 838.0) ^b^
MI	162.5 (144.3, 181.7) ^a^	132.4 (113.7, 154.8) ^b^	112.6 (101.2, 126.0) ^b^	182.4 (158.5, 208.3) ^c^
NH	440.7 (406.9, 474.0) ^a^	486.3 (433.5, 543.2) ^ab^	459.5 (418.9, 502.6) ^ab^	543.3 (488.4, 600.8) ^b^
ND	29.0 (26.1, 32.3) ^a^	-	26.0 (24.0, 28.2) ^b^	-
OR	22.8 (17.8, 27.7) ^a^	22.4 (18.0, 26.9) ^a^	23.1 (18.5, 27.7) ^a^	23.8 (18.9, 29.1) ^a^
RI	126.2 (115.8, 137.0) ^a^	109.0 (99.2, 119.0) ^b^	101.3 (91.6, 111.3) ^b^	131.6 (118.0, 145.1) ^a^
SC	30.7 (28.2, 33.5) ^a^	29.6 (27.2, 32.3) ^a^	29.3 (26.8, 31.8) ^a^	35.9 (32.4, 39.5) ^b^
TX	38.8 (36.5, 40.8) ^a^	37.5 (36.3, 38.7) ^a^	36.0 (34.2, 37.7) ^b^	44.5 (40.9, 47.9) ^c^
VA	1286.4 (1226.3, 1348.9) ^a^	1088.8 (1024.4, 1157.4) ^b^	1016.0 (966.7, 1067.4) ^c^	1375.1 (1292.3, 1462.4) ^d^
VT	204.6 (185.3, 224.5) ^a^	-	188.4 (167.1, 212.5) ^a^	-
WA	1.7 (1.4, 2.0) ^a^	1.8 (1.4, 2.2) ^a^	1.9 (1.5, 2.4) ^a^	1.9 (1.5, 2.3) ^a^
WV	116.5 (96.8, 141.2) ^a^	83.5 (75.9, 92.5) ^b^	80.6 (72.6, 89.2) ^b^	141.6 (114.6, 172.9) ^c^
Overall	248.4 (229.0, 266.9) ^a^	282.3 (259.5, 304.8) ^b^	221.5 (204.9, 238.3) ^c^	330.5 (304.4, 355.8) ^d^

Within a row, values sharing the same superscript letter are not significantly different (*p* > 0.05). ^1^ This model included human search terms + human case counts + canine search terms + environmental data.

**Table 6:** Mean Absolute Percentage Error (MAPE) and 95% bootstrapped CI of predictive models for monthly state-level canine Lyme disease case count (N = 1920).

	MAPE (%) (95% CI)			
State	Human Data (Search Terms + Cases)	Canine Search Terms	Environmental Data	One Health Data
CA	1390.1 (1168.4, 1672.4)	1400.2 (1165.3, 1692.3)	1390.1 (1153.9)	1438.2 (1208.7, 1732.6)
CT	838.4 (625.3, 1105.0)	862.9 (660.0, 1107.9)	791.7 (603.1, 1027.1)	847.9 (643.1, 1101.8)
IN	3693.2 (2972.0, 4517.2)	2955.0 (2297.4, 3771.8)	2712.3 (2166.3, 3381.5)	4232.9 (3358.4, 5283.4)
KS	158.9 (122.1, 197.2)	-	152.2 (118.1, 189.1)	-
ME	1198.5 (1020.6, 1395.9)	1233.2 (1042.7, 1445.3)	1283.7 (1054.8, 1525.1)	1411.9 (1170.1, 1677.4)
MI	3047.1 (2340.4, 3781.8)	2430.4 (1926.7, 2971.9)	2596.0 (1990.8, 3217.9)	3471.1 (2596.7, 4414.8)
NH	1626.1 (923.7, 2580.2)	1549.9 (914.7, 2357.7)	1541.7 (924.4, 2341.3)	1617.5 (997.7, 2391.4)
ND	1358.4 (1102.2, 1618.5)	-	1299.1 (1067.0, 1557.7)	-
OR	89.2 (64.8, 121.1)	99.4 (73.5, 132.4)	95.2 (70.7, 127.6)	79.8 (60.6, 109.7)
RI	269.8 (225.6, 318.1)	261.9 (218.1, 309.7)	240.5 (198.9, 285.2)	269.1 (216.3, 324.7)
SC	1074.8 (909.6, 1242.7)	1087.7 (907.0, 1274.7)	1080.6 (897.5, 1266.6)	1282.7 (1081.7, 1512.8)
TX	1468.5 (1106.9, 1842.2)	1401.1 (1083.0, 1721.9)	1341.9 (1033.9, 1646.8)	1680.6 (1249.3, 2154.3)
VA	3124.2 (2090.9, 4473.8)	2991.3 (2111.5, 4786.4)	2821.0 (1890.7, 4041.6)	3385.9 (2247.0, 4855.4)
VT	796.3 (654.6, 961.0)	-	814.7 (630.8, 1016.2)	-
WA	61.3 (48.3, 74.2)	59.2 (49.0, 72.4)	62.5 (51.7, 74.3)	74.2 (56.6, 94.8)
WV	916.7 (698.2, 1165.6)	868.2 (653.5, 1118.8)	913.3 (657.8, 1198.0)	1043.7 (776.1, 1338.4)
Overall	1280.6 (1161.2, 1412.2)	1313.8 (1189.2, 1445.5)	1195.5 (1092.1, 1318.4)	1549.3 (1396.9, 1722.4)

## Data Availability

Restrictions apply to the availability of these data. Data were obtained from IDEXX Laboratories and state health departments (see Supplementary File for more information).

## Supplementary Materials

The following supporting information can be downloaded at https://www.mdpi.com/article/10.3390/pathogens15040393/s1.

## Abbreviations

The following abbreviations are used in this manuscript:

MAE	Mean Absolute Error
MAPE	Mean Absolute Percentage Error
