# Supplementary material for: Predicting Lyme Disease: A One Health Approach
Source: Pathogens. 2026 Apr 7;15(4):393. doi: 10.3390/pathogens15040393 (PMC13118596; doi:10.3390/pathogens15040393)
Supplement: Supplementary file 1 [file pathogens-15-00393-s001.zip › pathogens-4196120-supplementary.pdf]

Data notes and sources

California:

Data notes from health department:
1) Lyme disease, like most infectious diseases, is probably under-reported. The CDC estimates that nationally, Lyme disease is under-reported by a factor of 10. We have not evaluated this specifically in California but understand that the reported cases that fit surveillance criteria does not reflect all cases.
2) The cases we include are those that fit surveillance criteria at that time for Confirmed or Probable.
3) The cases are included based on date of report since we have complete data for that; date of onset may be missing or can be as much as two years prior to date of report if case diagnosed later in course of disease.

Data source:
California Health and Human Services. Personal communication. Email correspondence. February 3, 2022.

Connecticut:

Data notes:
N/A

Data source:
Connecticut Department of Public Health. Lyme disease statistics. Accessed February 9, 2022.
https://portal.ct.gov/DPH/Epidemiology-and-Emerging-Infections/Lyme-Disease-Statistics

Indiana:

Data notes:
N/A

Data source:
Indiana Department of Health. Indiana report of infectious diseases. Accessed June 20, 2022.
https://www.in.gov/health/health-and-human-services/indiana-report-ofinfectious-diseases/

Michigan:

Data notes from health department:
All confirmed and probable cases from 2011-2020 that were entered in the state electronic surveillance system, MDSS were extracted. If onset date was blank, date of diagnosis was used, and if that was also blank, report date, the date the case was reported to the health department, was used). Please note that because of the methodology used for assigning a date to each case, these numbers are very slightly different from the annual total numbers reported previously (such as on the MI Disease Mapper).

Data source:
Michigan Department of Community Health. Personal communication. Email correspondence. March 4, 2022.

New Hampshire:

Data notes from health department:
There are some years between 2010 and 2021 that the Lyme Disease surveillance was not able to be completed in full. These years are namely 2015, 2016, 2020 and 2021. Some of these are more complete years then others (e.g. for 2015 and 2016 compared to 2020 and 2021, when a larger portion of the reports were reviewed), but nonetheless these are all considered incomplete years and case counts/rates should be viewed as partial.

Data source:
New Hampshire Department of Health and Human Services. Personal communication. Email correspondence. April 27, 2022.

Oregon:

Data notes:
N/A

Data source:
Oregon Health Authority. Annual communicable disease reports. Accessed June 10, 2022.
https://www.oregon.gov/oha/PH/DISEASESCONDITIONS/COMMUNICABLEDISEASE/DISEASESURVEILLANCEDATA/ANNUALREPORTS/Pages/arpt.aspx

Rhode Island:

Data notes from health department:
Data for this request have been extracted from the Rhode Island National Electronic Disease Surveillance System and filtered by event date on February 3, 2022. In 2013, Rhode Island enhanced its Lyme disease surveillance system; therefore, the dramatic increase in reported disease is due to increased surveillance rather than increased illness. Rhode Island Lyme disease case counts for surveillance year 2021 are preliminary data as of February 2, 2022. Rhode Island surveillance data for 2022 has not been finalized and the data is subject to change.

Data source:
Rhode Island Department of Health. Personal communication. Email correspondence. February 11, 2022.

Texas:

Data notes from health department:
The Lyme disease case counts are by residence county, not county of disease acquisition.

Data source:
Texas Department of State Health Services. Personal communication. Email correspondence. June 6, 2022.

Vermont:

Data notes from health department:
To calculate monthly Lyme disease cases, the earliest of three dates in this order was used: symptom onset date, diagnosis date, report date (date case/lab result was reported to the VT Dept of Health, NOT lab report date as previously written). The earliest date of those three dates was then used to assign a month to the case. This mirrors the hierarchy of dates the

Dept. of Health regularly uses to assign MMWR weeks to each case.

Data source:
Vermont Department of Health. Personal communication. Email correspondence. March 1, 2022.

Virginia:

Data notes:
N/A

Data source:
Virginia Department of Health. Virginia reportable disease surveillance data. Accessed June 20, 2022. https://www.vdh.virginia.gov/surveillance-and-investigation/virginiareportable-disease-surveillance-data/
